# Supplementary material for: The Role of mRNA Alternative Splicing in Macrophages Infected with Mycobacterium tuberculosis: A Field Needing to Be Discovered
Source: Molecules. 2024 Apr 16;29(8):1798. doi: 10.3390/molecules29081798 (PMC11052237; doi:10.3390/molecules29081798)
Supplement: Supplementary file 1 [file molecules-29-01798-s001.zip › molecules-2757998-supplementary.pdf]

**Gene**	**Response to *Mtb* infection**
RAB8B	RAB8B truncated variant is enriched in H37Rv-infected macrophages.
ABCC2	ABCC2 were upregulated in macrophages infected with M. tuberculosis.
TNFAIP2	Generate TB-specific alternative splicing isoforms.
SLC26A11	Generate TB-specific alternative splicing isoforms.
IFI27	Generate TB-specific alternative splicing isoforms.
IFIT3	Generate TB-specific alternative splicing isoforms.
OAS1	Generate TB-specific alternative splicing isoforms.
OASL	Generate TB-specific alternative splicing isoforms.
GNB1	Generate TB-specific alternative splicing isoforms.
TNFAIP2	Generate TB-specific alternative splicing isoforms.
S100A8-intron1-retention intron	Generate TB-specific alternative splicing isoforms.
RPS20-exon1 alternative promoter	Generate TB-specific alternative splicing isoforms.
KIF13B-exon4-skipping exit	Generate TB-specific alternative splicing isoforms.
UBE2B-exon7-SE	Generate TB-specific alternative splicing isoforms.
SRSF2	After infection with H37Rv, the expression levels of SRSF2 were significantly downregulated.
SRSF3	After infection with H37Rv, the expression levels of SRSF3 were significantly downregulated.
SRp75	After infection with H37Rv, the expression levels of SRp75 were significantly downregulated.
SF3a	After infection with H37Rv, the expression levels of SF3a were significantly downregulated.
IL-4	The mRNA level of IL-4 expression was higher in unstimulated peripheral blood mononuclear cells of tuberculosis patients. Expression of IL-4δ2 was increased in latently infected healthy TB contacts.
IL7	There are nine isoforms of IL7, including IL7c, IL7δ3, IL7δ4, IL7δ5, IL7δ3/4, IL7δ4/5, IL7δ3/4/5, IL7δ3/4-52bp E2, and IL7δ4/5-52bp E2. In peripheral blood monocytes of non-human primates, the highest expression level is IL-7c. IL-7δ5 and IL-7δ4/5 were mainly expressed in the granuloma tissue from patients with latent tuberculosis.
IL-7R	IL-7R has three isoforms, namely IL-7Rc, IL-7Rδ6, and IL-7Rδ5/6. sIL-7R acts as a isoform that inhibits IL-7 activity, and appears to have the opposite effect to full-length IL-7Rc.
IL12-Rβ1	IL12-Rβ1 is extremely important for humans to resist various intracellular pathogens, including Mtb.
IL-12Rβ1ΔTM	The role of IL-12Rβ1ΔTM is to enhance IL-12Rβ1-dependent dendritic cells (DCs) migration and activate Mtb-specific T cells. Mice lacking IL-12Rβ1ΔTM will have an impact on their ability to control infection with Mtb in extrapulmonary lung organs.
IL-32	IL-32 has nine isoforms, namely IL-32 α, IL-32 β, IL-32 γ, IL-32 δ, IL-32 ε, IL-32 ζ, IL-32 η, IL-32 θ, and IL-32s. The expression level of IL-32 in patients with pulmonary tuberculosis was lower than that in healthy people. Compared with mouse macrophages expressing IL-32γ and IL-32β, macrophages expressing IL-32γ alone were more effective in limiting the growth of Mtb

Table S1: Changes in alternative splicing after macrophage infection by *Mycobacterium tuberculosis.*
